# Partial Atresia of the Urethra

**Published:** 1899-10

**Authors:** W. H. Heath

**Affiliations:** Buffalo, N. Y., Clinical professor genito-urinary diseases, Medical Department, University of Buffalo, surgeon genito-urinary department, Erie County Hospital, etc.


### Clinical Report.

### PARTIAL ATRESIA OF THE URETHRA.

By W. H. HEATH, M. D., Buffalo, N. Y.,
Clinical professor genito-urinary diseases, Medical Department, University of Buffalo, surgeon genito-urinary department, Erie County Hospital, etc.

W. N., EIGHTEEN hours after birth, May 11, 1899, the nurse in attendance reported that although the child's bowels had acted freely, it had passed no urine, and that the infant was irritable, crying almost incessantly as though in pain and suffering. Examination showed the diminutive penis to be semi-erect, rather swollen and tender on manipulation; a prepuce very elongated with aperture and passage through it so small and tortuous as not to admit the smallest pocket-case probe.

A circumcision was at once made, which on completion revealed further a rudimentary pin-hole meatus with an imperforate urethra, forming a cul-de-sac about half inch in depth, evidently due to an agglutination of the walls of the urethra. The meatus was carefully incised and the urethral obstruction forced with a delicate, sharp pointed tenotome and dilated with thin bladed forceps. This accomplished, it was fully expected that a flow of urine would ensue as the bladder was so distended as to be evident through the abdominal walls, while the urethra, under the scrotum, was distinctly bulging. Urine not appearing, a catheter was passed, which determined that a second obstruction existed at about the peno-scrotal juncture. This was carefully subjected to the same procedure, when a gush of urine followed. Since that time micturition has been normal and uneventful. The first urethral obstruction was apparently one quarter of an inch in depth, quite fibrous and resisting; the deeper one less so in character. No deleterious effect upon the health of the child was noticeable nor have the urinary organs themselves apparently suffered.

The interesting features of the case are that it is one of rarity and that the urinary organs did not suffer, so far as can be determined by their subsequent action. This may be taken that the condition was recent and indicated the cause as pathological, rather than a malformation. Further, that the procedures were successful. Successfully cutting through an obstruction in tissues so delicate and anatomically difficult by diminutive size, etc., without the aid of sight, with no misadventure, perforation of urethra, etc., is good fortune. Concerning urination by the fetus in utero, there is yet some question. That the function is performed and is essential, though irregularly performed, is evinced by the chemical character of amniotic fluid and the disturbances noted in a few recorded cases of complete atresia. In these cases dilatation of everything back of the point of obstruction follows and has been noted to such an extent as to be an impediment to delivery.

Its cause is not clear. It may be a vice of development, but is most likely pathological. An extremely acid or irritative urine with susceptibility of tissue readily causes irritation, inflammation and with the new delicate fetal mucous membrane added adhesions of opposite surface would be a rational explanation.

The possibility of suppression and mechanical obstruction being easily confounded in infants justifies that in all cases of delayed urination that a soft rubber catheter be passed at once to determine this point, as congenital malformation and obstruction are more likely than suppression, which is pathologically rare in an infant.

As to how long nonurination in an infant should be permitted, it can be summed up by saying it should not be tolerated at all. If when the bowels act the bladder is not emptied its delay and cause should be accounted for at once and treatment instituted.

131 FRANKLIN STREET.

In view of the question raised by Dr. Heath in the above article, the following editorial from the *New York Medical Journal* of September, will be of interest.—[ED.]

### THE LIQUOR AMNII AND THE FETAL KIDNEYS.

It has long been a matter of discussion as to whether or not the kidneys of the fetus were functionally active, and consequently, as to whether or not the liquor amnii consisted in great part of the urine of the fetus. To settle the question, Schaller (*Archiv. für Gynäkologie*, lvii, 3; *Deutsche Medizinal-Zeitung*, April 24th,) has resorted to a most ingenious method of investigation—namely, that of administering phlorrhizin to pregnant women. Phlorrhizin, a constituent of the root bark of various fruit trees, has been known for years to have the property of causing glycosuria when ingested. Schaller's plan was to ascertain if the liquor amnii contained sugar in considerable amount after the administration of phlorrhizin to the mother. If he found none, he would conclude that the *fetus in utero* did not urinate; if he found only a small amount, he would infer that the functional activity of the fetal kidneys was displayed only during the late period of intrauterine life; if he found it in abundance, he would agree with Gusserow and his followers that the liquor amnii was largely composed of fetal urine. All this was founded on the assumption that phlorrhizin administered to the mother would pass into the fetal circulation and be carried to the fetal kidneys, and it is in the kidneys that the sugar of phlorrhizin glycosuria is wholly or for the most part formed.

Schaller concludes that there are no regular secretion and periodical excretion of urine by the fetus, even at the very end of pregnancy; that the functional activity of the fetal kidneys does not begin until the process of parturition induces changes in the placental circulation, and that even during labor the fetus does not generally urinate into the liquor amnii; that it is only very exceptionally that the fetal bladder is emptied into the liquor amnii, and then most probably in consequence of disturbances of the fetal circulation; that the liquor amnii is a transudation from the maternal vessels; and that the kidneys of the newborn child perform their function more slowly than those of the adult. These observations seem to settle the question of the relationship of the liquor amnii to the urine of the fetus.

